# Physiological role for leptin in the control of thermal conductance

**DOI:** 10.1016/j.molmet.2016.07.005

**Published:** 2016-07-20

**Authors:** Karl J. Kaiyala, Kayoko Ogimoto, Jarrell T. Nelson, Kenjiro Muta, Gregory J. Morton

**Affiliations:** 1 UW Diabetes Institute, Department of Medicine, University of Washington, Seattle, WA, 98109, USA; 2 Department of Oral Health Sciences, School of Dentistry, University of Washington, Seattle, WA, 98195, USA

**Keywords:** Thermoregulation, Thermal conductance, Energy expenditure, Energy intake, Body temperature, Leptin, sc, subcutaneous, BAT, brown adipose tissue, Ucp1, uncoupling protein-1, DIO, diet-induced obesity

## Abstract

**Objective:**

To investigate the role played by leptin in thermoregulation, we studied the effects of physiological leptin replacement in leptin-deficient *ob/ob* mice on determinants of energy balance, thermogenesis and heat retention under 3 different ambient temperatures.

**Methods:**

The effects of housing at 14 °C, 22 °C or 30 °C on core temperature (telemetry), energy expenditure (respirometry), thermal conductance, body composition, energy intake, and locomotor activity (beam breaks) were measured in *ob/ob* mice implanted subcutaneously with osmotic minipumps at a dose designed to deliver a physiological replacement dose of leptin or its vehicle-control.

**Results:**

As expected, the hypothermic phenotype of *ob/ob* mice was partially rescued by administration of leptin at a dose that restores plasma levels into the physiological range. This effect of leptin was not due to increased energy expenditure, as cold exposure markedly and equivalently stimulated energy expenditure and induced activation of brown adipose tissue irrespective of leptin treatment. Instead, the effect of physiological leptin replacement to raise core body temperature of cold-exposed *ob/ob* mice was associated with reduced thermal conductance, implying a physiological role for leptin in heat conservation. Finally, both leptin- and vehicle-treated *ob/ob* mice failed to match energy intake to expenditure during cold exposure, resulting in weight loss.

**Conclusions:**

The physiological effect of leptin to reduce thermal conductance contributes to maintenance of core body temperature under sub-thermoneutral conditions.

## Highlights

• Physiological leptin replacement partially rescues hypothermia in cold-exposed *ob/ob* mice.
• Leptin's normothermic effect cannot be explained by increased energy expenditure.
• This effect does not appear to be mediated by changes in physical activity.
• Leptin promotes normothermia during cold exposure by reducing thermal conductance.

## 1 Introduction

The ability to regulate core body temperature within normothermic limits across a wide range of environmental temperatures is a defining characteristic of all mammals. Free-living animals are routinely exposed to thermal environments that can change rapidly and dramatically on both a daily and seasonal basis, and the energy needs associated with defense of core body temperature in these changing environments can be substantial. Owing to their greater surface-area to-body mass ratio and low thermal inertia, the challenge posed by a cool environment on small mammals is far greater than in larger endothermic animals in terms of the need for both heat production and retention [1]. Unless the energy homeostasis system can meet the challenges imposed by changing thermogenic needs, the amount of body fuel stored as adipose tissue will fluctuate dramatically. In response to chronic cold exposure, for example, the rate of energy expenditure must increase sharply to generate the additional heat needed to avert a drop in core body temperature, and unless energy intake increases proportionately, a progressive decline in body fat mass will result. Simultaneously, the efficiency with which body heat is retained must also increase if core body temperature is to be maintained in a cold environment [1]. Living in a warm environment poses the opposite challenge, as a declining need for heat production will ultimately cause weight gain and increased body temperature unless the decline in thermogenesis is offset by the combined effects of reduced energy intake and an increased rate of heat dissipation to the environment (thermal conductance).

A growing body of evidence suggests that leptin plays a key role in not only the regulation of energy homeostasis but thermoregulation as well [2]. For example, leptin-deficient *ob/ob* mice are characterized not only by hyperphagia and obesity, but also mild hypothermia when housed at room temperature, and they become profoundly hypothermic when exposed directly to cold environments [3], [4], [5], [6]. Surprisingly, this thermoregulatory failure is not due to a failure to increase energy expenditure in response to changing ambient temperatures, as they do so in a manner that resembles the response of wild-type mice [7], [8]. To explain this paradoxical finding, a recent report suggested that the mechanism underlying leptin's effect to raise defended body temperature involves an increase of the body temperature threshold for activating thermogenesis rather than a stimulatory effect on thermogenesis *per se* [9]. An alternative possibility is that leptin plays a physiological role to limit thermal conductance, which describes the ease with which heat flows from the body core to the environment and is an essential aspect of mammalian thermoregulation [1], [10], [11], [12]. According to this hypothesis, hypothermia in *ob/ob* mice is predicted to be secondary in part to their failure to reduce thermal conductance as a consequence of leptin deficiency. A major goal of the current work was to test this hypothesis.

In addition, whereas *ob/ob* mice increase energy expenditure appropriately in response to a cold challenge, they are unable to adjust energy intake to meet changing energy needs associated with housing across a range of temperatures and consequently lose excessive weight in the cold, while gaining excessive weight in warm environment [7], [8], [13]. Thus, adaptive changes of energy intake and thermal conductance, but not energy expenditure, appear to require intact leptin signaling [8].

To further clarify the physiological role played by leptin in both thermoregulation and the coupling of thermoregulatory needs to adaptive changes in energy homeostasis, leptin-deficient *ob/ob* mice were infused subcutaneously with either vehicle or a dose of leptin designed to restore plasma levels to those of wild-type controls, and were housed at ∼thermoneutrality (30 °C), room temperature (22 °C), or at a cool temperature (14 °C). During this time, comprehensive measures of energy balance and core temperature were continuously obtained and measures of thermal conductance determined.

## 2 Materials and methods

### 2.1 Animals

Adult male C57/Bl6 mice and leptin-deficient *ob/ob* mice on the C57/Bl6 background strain were obtained from Jackson Laboratories, ME. All studied animals were individually housed in a temperature-controlled room with a 12:12 h light:dark cycle under specific-pathogen free conditions and provided with *ad libitum* access to water and chow unless otherwise stated (PMI Nutrition, MO). All procedures were performed in accordance with NIH Guidelines for the Care and Use of Animals and were approved by the Animal Care Committee at the University of Washington.

### 2.2 Systemic leptin administration

Adult male *ob/ob* mice were separated into weight-matched groups, respectively and implanted subcutaneously (sc) with an osmotic minipump (Alzet Model 1007D; DURECT Corporation, Cupertino, CA) containing either vehicle (PBS; pH 7.9) or leptin at a dose of 100 ng/h (Dr. A.F. Parlow; National Hormone & Peptide Program, CA) (n = 7–8 per group) designed to achieve leptin levels in the physiological range for wild-type mice based on previous studies [14].

### 2.3 Core temperature

Adult male *ob/ob* mice underwent implantation of body temperature transponders into the peritoneal cavity (Starr Life Science Corp, Oakmont, PA). Following at least a one-week recovery period, animals were acclimated to metabolic cages enclosed in temperature- and humidity-controlled cabinets (Caron Products and Services, Marietta, OH). Signals emitted by body temperature transponders were sensed by a receiver positioned near the cage and analyzed using VitalView software as previously described [8].

### 2.4 Measurements of energy expenditure, food intake and ambulatory activity

Animals were acclimated to indirect calorimetry cages prior to study and data collection. Energy expenditure measures were determined using a computer-controlled indirect calorimetry system (Promethion^®^, Sable Systems, Las Vegas, NV) with support from the University of Washington Nutrition Obesity Research Center (NORC) Energy Balance and Glucose Metabolism (EBGM) Core as described in detail previously [8], [15]. The calorimetry system consists of 16 metabolic cages (similar to home cages with bedding) that are equipped with water bottles and food hoppers connected to load cells for continuous food and water intake monitoring and housed in a temperature- and humidity-controlled cabinet (Caron Products and Services, Marietta, OH). O_2_ consumption and CO_2_ production were measured for each animal for 1 min at 10-min intervals. Respiratory quotient (RQ) was calculated as the ratio of CO_2_ production to O_2_ consumption. Energy expenditure was calculated using the Weir equation [16]. Whole body thermal conductance was calculated as energy expenditure divided by the difference between core and ambient temperature [1], [8]. Ambulatory activity was determined simultaneously with the collection of calorimetry data. Consecutive adjacent infrared beam breaks in the x-, y- and z-axes were scored as an activity count, and a tally was recorded every 10 min. Data acquisition and instrument control were coordinated by MetaScreen v.1.6.2 and raw data was processed using ExpeData v.1.4.3 (Sable Systems) using an analysis script documenting all aspects of data transformation.

### 2.5 Thermal conductance

To quantify whether leptin regulates the ease with which heat flows from the body core to the environment (via convection, conduction, radiation and evaporation), we calculated whole body thermal conductance of *ob/ob* mice receiving either vehicle or leptin at a dose intended to achieve physiological leptin replacement. Thermal conductance is a useful parameter in comparative thermoregulatory studies of rodents [12], [17] and is derived from the formula: C = EE/(Tb − Ta), where C = conductance; EE = energy expenditure; Tb = core temperature and Ta = ambient temperature [1], [8], [10], [11], [12], [17]. This parameter of heat transfer has long been calculated based on energy expenditure in situations where the mean energy expenditure rate equals the mean heat loss rate as is true over time intervals that involve very little or no net change in core temperature (e.g., 24 h periods) [8].

### 2.6 Body composition analysis

In a separate cohort of adult male *ob/ob* mice that were not implanted with body temperature transponders, measures of body fat mass and lean mass were determined using quantitative magnetic resonance spectroscopy (QMR) (EchoMRI 3-in-1; Echo MRI, TX) using the University of Washington NORC EBGM Core [18].

### 2.7 Experimental protocol

After acclimation to cages situated within temperature- and humidity-controlled chambers, to allow for pair-wise comparisons, measures of energy expenditure, food intake, locomotor activity and body temperature were recorded continuously in all mice for 68 h with the temperature maintained at 22.0 ± 0.1 °C before being implanted with a minipump infusing either vehicle or leptin as described above. These same measures were then recorded in animals that were housed at either 30.0 ± 0.1 °C (a temperature that is, or is close to, thermoneutrality in WT mice) [1], [19], 22.0 ± 0.1 °C or to 14.0 ± 0.1 °C. Temperature changes occurred gradually over a 4-h interval and mice remained at each temperature for 68 h. A separate group of *ob/ob* mice were subjected to the same protocol described above except that they were not implanted with body temperature transponders to permit measures of body composition.

### 2.8 Blood collection and tissue processing

At study completion, brown adipose tissue (BAT) samples were harvested and subsequently stored at −80 °C for further analysis. Whole blood was collected into EDTA-treated tubes and centrifuged, and plasma was removed and stored at −80 °C for subsequent assay. Plasma leptin levels were determined by ELISA (Crystal Chem, Chicago, IL).

### 2.9 RT-PCR

Total RNA was extracted from BAT using TRIzol according to manufacturer's instructions (MRC, Cincinnati, OH). RNA was quantitated by spectrophotometry at 260 nm (Nanodrop 1000; Thermo Scientific, DE) and reverse-transcribed with AMV reverse transcriptase (1 μg) (Promega, Madison, WI), and real-time PCR was performed on a ABI Prism 7900 HT (Applied Biosystems) using the commercially available PCR master mix (SYBR Green, Applied 2.0; Applied Biosystems), as described previously [20]. PCR data were analyzed using the Sequence Detection System software (SDS Version 2.2; Applied Biosystems). Expression levels of each gene were normalized to a housekeeping gene (18S RNA) and expressed as a percentage of controls. Non template controls were incorporated into each PCR run.

### 2.10 Statistical analysis

Results are expressed as mean ± SEM. Significance was established at p < 0.05, two tailed. Our design focused on directional planned comparisons between leptin and vehicle-treated mice based on the scientific hypotheses that leptin treatment would increase energy expenditure and decrease thermal conductance at sub-thermoneutrality [8] and promote a state of negative energy balance [21], [22], [23]. For statistical comparisons involving core temperature, energy expenditure, ambulatory activity, RQ or food intake, data obtained during the 14 °C, 22 °C and 30 °C test periods were reduced into mean light and dark photoperiod components and into 24 h means for each mouse. Statistical analyses were performed using Statistica (version 7.1; StatSoft, Inc., Tulsa, OK) and SPSS (version 23, IBM Corp., Somers, NY). A mixed factorial (group by ambient temperature) ANOVA with a least significant difference pairwise test was used to compare mean values between groups in analyses that did not entail adjustment for body size. Analyses that were adjusted for body size were performed with the mixed model method in SPSS. A two-sample unpaired Student's t-test was used for two-group comparisons and a paired t-test for within group comparisons.

## 3 Results

### 3.1 Effect of physiological leptin replacement on core body temperature at different ambient temperatures

**Figure 1:**
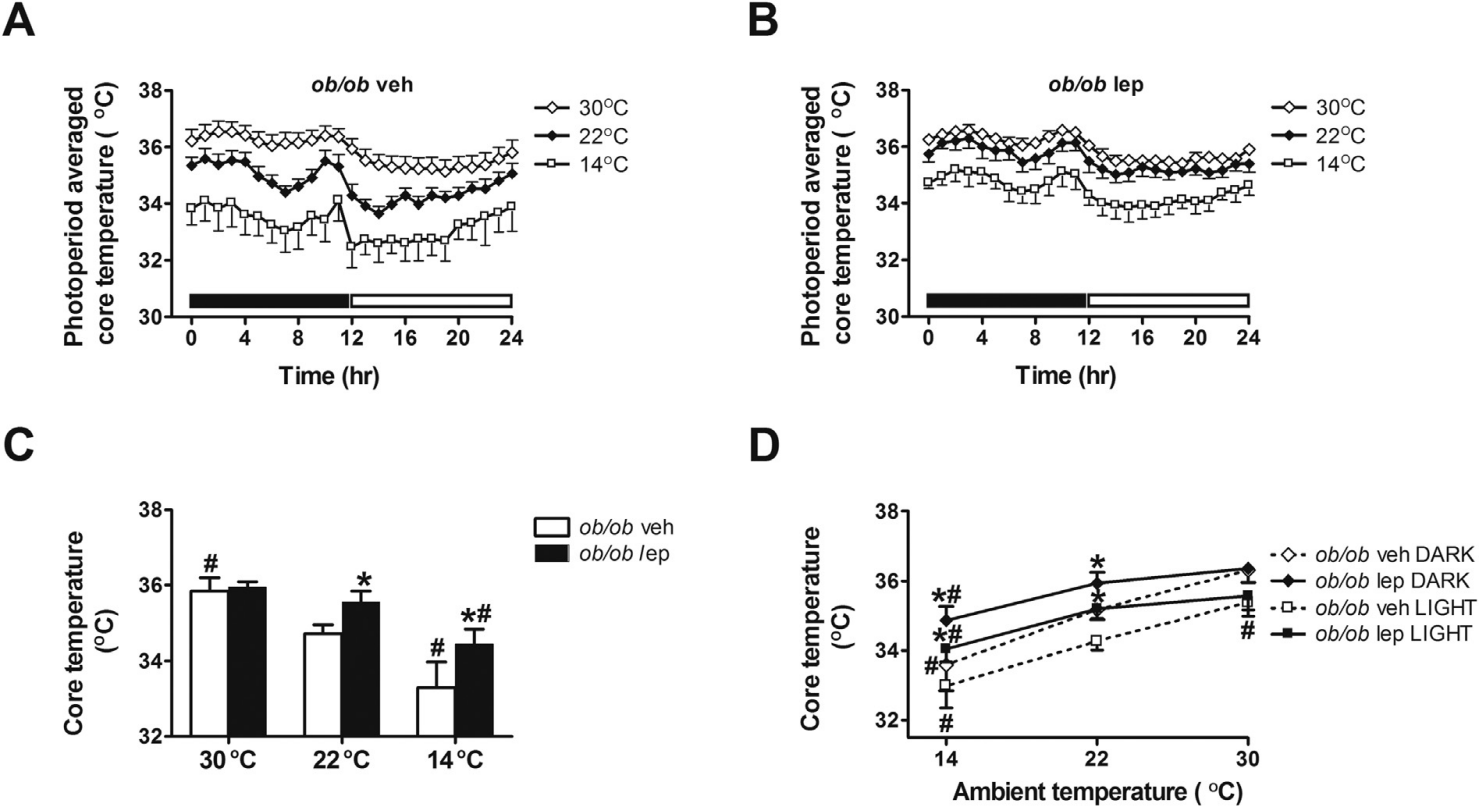
**Effect of leptin treatment on core body temperature at different ambient temperatures**. (**A, B**) Photoperiod averaged core body temperature, (**C**) mean daily core body temperature and (**D**) the relationship between core body temperature and ambient temperature during the dark cycle and light cycle in adult male *ob/ob* mice treated with either vehicle or a physiological replacement dose of leptin administered systemically under different ambient temperature conditions (n = 5–6/group). Solid bar represents dark cycle; Open bar represents light cycle. Mean ± SEM. *p < 0.05 vs. *ob/ob* veh; #p < 0.05 vs. 22 °C.

To investigate the physiological role of leptin in thermoregulation, leptin-deficient *ob/ob* mice received a continuous sc infusion of either vehicle or leptin and were subsequently housed in each of three different thermal environments. By design, the dose of leptin administered to these *ob/ob* mice at each temperature raised plasma leptin levels to values similar to those of wild-type mice (based on data from a previous study [8]: WT 22 °C: 1.72 ± 0.16 ng/ml vs. leptin-treated *ob/ob* mice: 30 °C, 2.56 ± 0.29; 22 °C, 2.00 ± 0.15; 14 °C, 2.32 ± 0.27 ng/ml). Whereas wild-type mice maintain normal body temperature under these conditions, our work replicates previous evidence [8], [19] that *ob/ob* mice fail to defend normothermia throughout each photoperiod when housed at 22 °C, despite exhibiting an intact diurnal temperature rhythm. While this hypothermia is not observed in *ob/ob* mice housed under ∼thermoneutral conditions (30 °C), it becomes even more pronounced when they are exposed to 14 °C (Figure 1). These findings confirm previous evidence linking leptin deficiency to defective thermoregulation when mice are directly housed at temperatures below thermoneutrality [4], [5], [6] without periods of cold-acclimation [4].

The thermoneutral zone is the range of ambient temperatures in which core temperature is maintained within normothermic limits at minimal metabolic expense via modulation of vasomotor tone, postural changes and hair/fur orientation such that basal metabolic rate is sufficient to maintain normothermia [24]. In both genetically normal and *ob/ob* mice 30 °C is a ∼thermoneutral temperature [1], [9]. At this temperature, *ob/ob* vehicle-treated mice maintained normal core body temperature and there was no effect of leptin on this outcome. This observation indicates that hypothermia in *ob/ob* mice is not secondary to an inherent inability to maintain normal body temperature; instead, the defect appears to involve a failure to either generate or retain a sufficient amount of heat to avert hypothermia when housed at sub-thermoneutral temperatures. At temperatures approximating thermoneutrality, therefore, leptin is not required for maintenance of normal core body temperature. At ambient temperatures of either 22 °C or 14 °C, by comparison, systemic leptin replacement raised mean core body temperature relative to vehicle-treated animals during both dark and light cycles (Figure 1). In sub-thermoneutral environments, therefore, leptin is required for intact thermoregulation, and in leptin-deficient mice, physiological leptin replacement is sufficient to raise core low body temperature towards the normal range.

A more detailed analysis of these body temperature responses reveals that under thermoneutral conditions, core temperature declines in vehicle-treated *ob/ob* mice during the first dark cycle and a similar pattern is seen in leptin-treated *ob/ob* mice (Supplemental Figure 1). When exposed to temperatures below thermoneutrality, however, we observed a modest but progressive increase in core body temperature over time in leptin-treated mice during both dark and light cycles, especially when housed at 14 °C (Mean Core Temperature Day 1: 34.03 ± 0.32 °C vs. Day 2: 34.38 ± 0.42 °C vs., Day 3: 34.72 ± 0.41 °C; p < 0.05 for each) (Supplemental Figure 1). This steady increase of core temperature over time, which was not observed in vehicle-treated *ob/ob* mice, raises the possibility that adaptive heat generating and/or conserving mechanisms are recruited over time once plasma leptin levels are raised into the physiological range. If so, it is possible that had studies been carried out over a longer period, core body temperature of leptin-replaced *ob/ob* mice would eventually have increased into the normal range.

### 3.2 Effect of physiological leptin replacement on energy homeostasis measured under different thermal conditions

**Figure 2:**
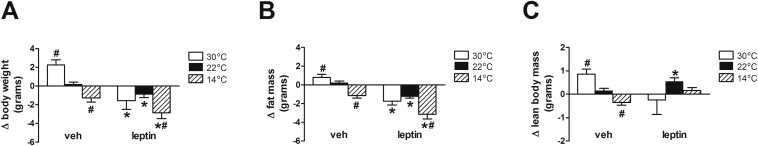
**Effect of leptin treatment on body composition at different ambient temperatures**. Change in (**A**) body weight, (**B**) fat mass and (**C**) lean body mass in adult male *ob/ob* mice treated with either vehicle or a physiological replacement dose of leptin administered systemically under different ambient temperature conditions (n = 5–6/group). Mean ± SEM. *p < 0.05 vs. *ob/ob* veh; #p < 0.05 vs. 22 °C.

To more fully characterize the physiological role of leptin in energy homeostasis in different thermal environments we also measured energy expenditure and energy intake in vehicle- and leptin-treated *ob/ob* mice housed at each of the three temperatures and, in a separate cohort of mice, measures of body composition were obtained as well. Relative to animals housed at 22 °C, vehicle-treated *ob/ob* mice exhibited excess weight gain when housed at 30 °C, an effect associated with increases of both fat and lean mass (Figure 2). When exposed to a colder ambient temperature (14 °C), these mice were unable to maintain neutral energy balance and hence exhibited excessive weight loss due to reductions of both fat and lean mass (Figure 2), consistent with a prior report [8]. By comparison, systemic leptin replacement induced weight loss owing to a selective loss of fat mass both at 30 °C and 22 °C. Leptin replacement also accentuated weight loss in *ob/ob* mice exposed to the cooler environment (14 °C) (Figure 2). Despite its ability to ameliorate hypothermia in *ob/ob* mice during cold exposure, therefore, physiological leptin replacement strongly promotes negative energy balance and weight loss in this setting, unlike that seen in wild-type mice [8].

**Figure 3:**
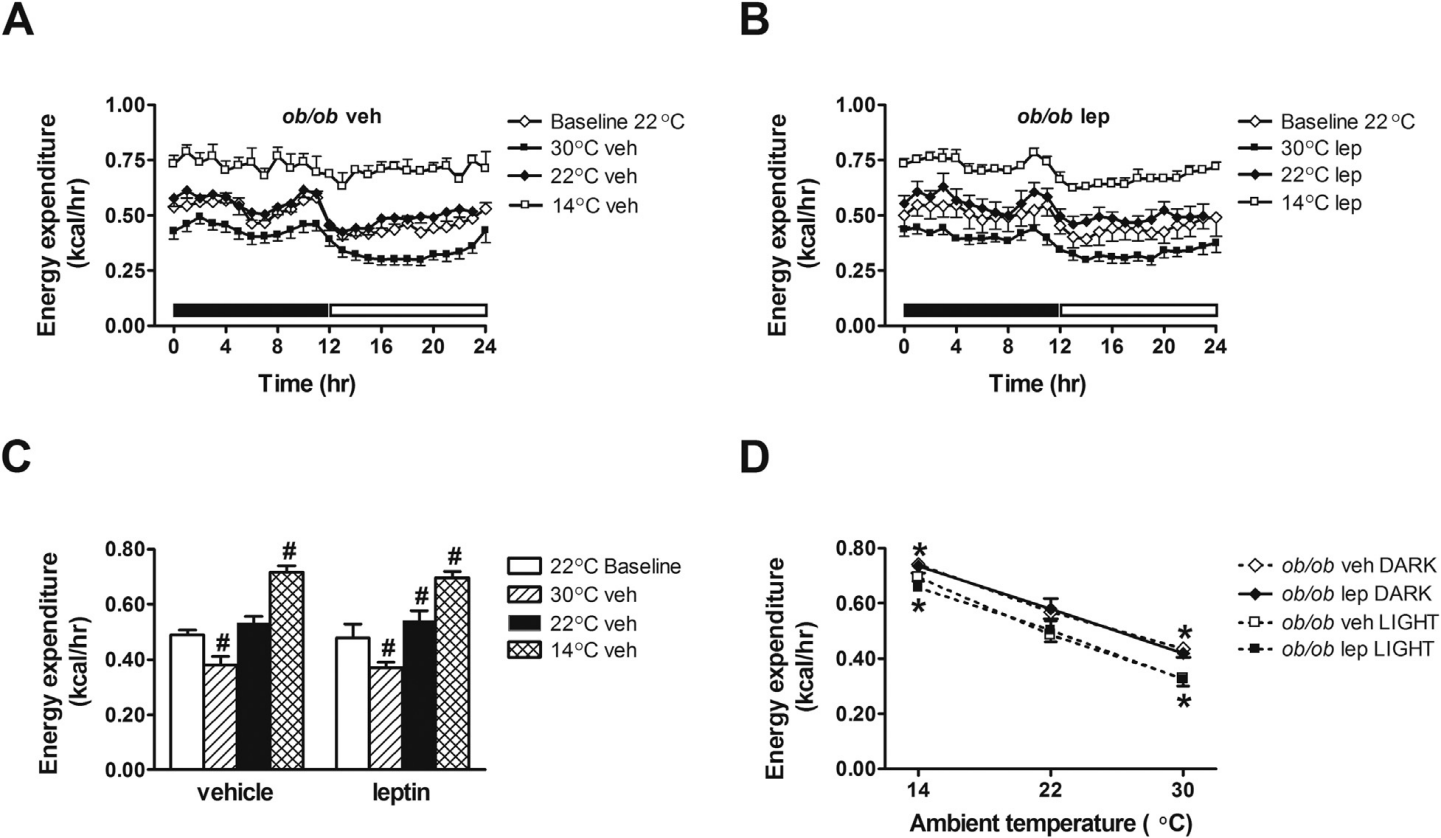
**Effect of leptin treatment on energy expenditure at different ambient temperatures**. (**A, B**) Photoperiod-averaged energy expenditure and (**C**) mean daily energy expenditure and (**D**) the relationship between energy expenditure and ambient temperature during the dark cycle and light cycle in adult male *ob/ob* mice at baseline (22 °C) and treated with either vehicle or a physiological replacement dose of leptin administered systemically under different ambient temperature conditions (n = 5–6/group). Solid bar represents dark cycle; Open bar represents light cycle. Mean ± SEM. #p < 0.05 vs. 22 °C Baseline. *p < 0.05 vs. 22 °C.

Consistent with previous observations [7], [8], [9], we found that hypothermia in cold-exposed *ob/ob* mice is not due to a failure to increase energy expenditure. On the contrary, energy expenditure increased robustly during both dark and light photoperiods in vehicle-treated *ob/ob* mice exposed to 14 °C, whereas a compensatory reduction of energy expenditure was evident when housed under approximately thermoneutral conditions (Figure 3). While we found that leptin increased energy expenditure at 22 °C during both the dark and light cycle relative to the baseline condition at this temperature (p < 0.05) (Figure 3), an effect not observed in vehicle-treated controls (p = ns), leptin-treated *ob/ob* mice exhibited a similar increase of energy expenditure relative to vehicle-treated mice when housed at 14 °C. Thus, leptin does not appear to be required for mice to adjust energy expenditure in response to changes of ambient temperature.

**Figure 4:**
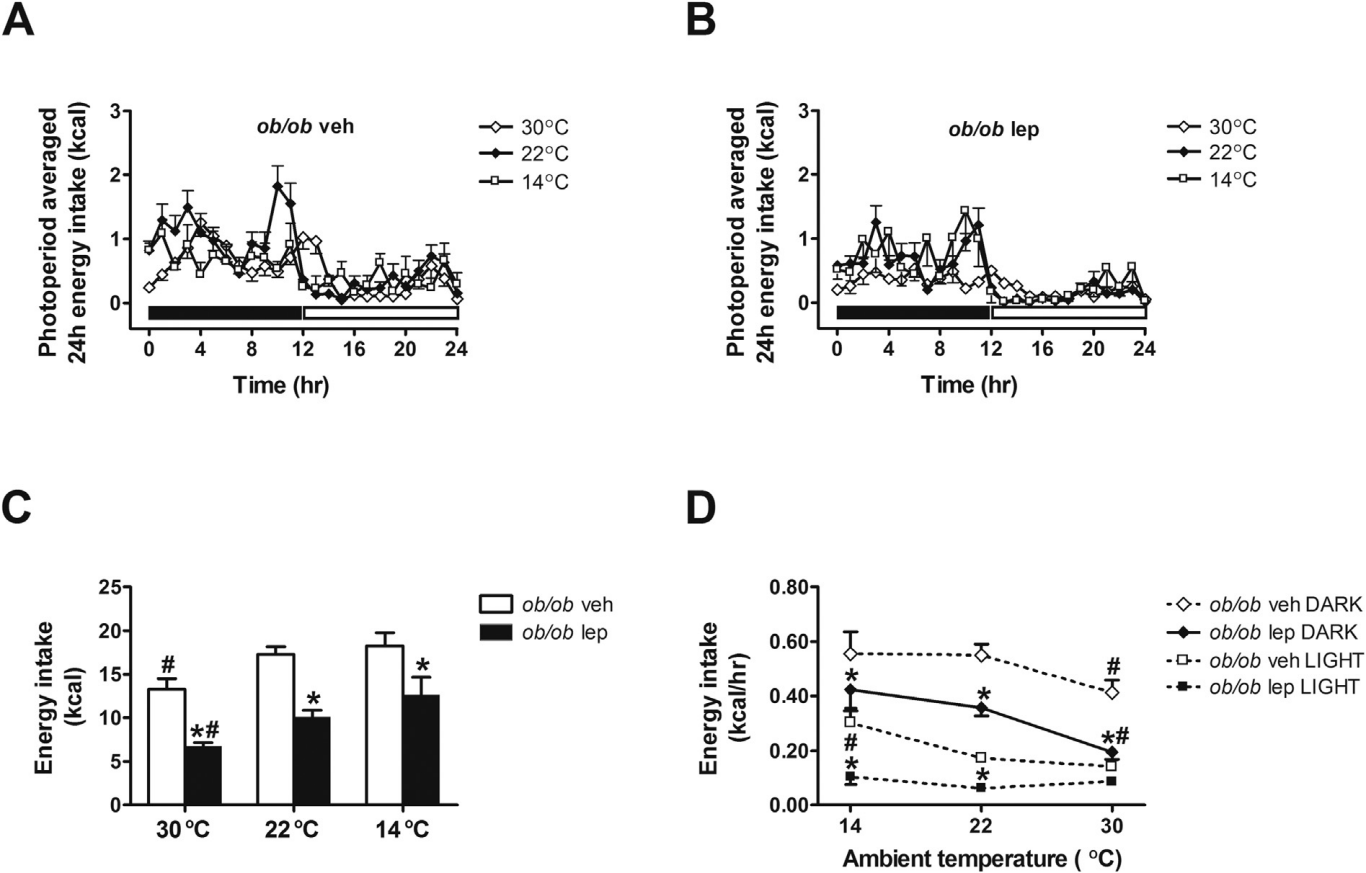
**Effect of leptin treatment on energy intake at different ambient temperatures**. (**A, B**) Photoperiod-averaged 24 h energy intake, (**C**) mean daily energy intake and (**D**) the relationship between energy intake and ambient temperature during the dark cycle and light cycle in adult male *ob/ob* mice treated with either vehicle or a physiological replacement dose of leptin administered systemically under different ambient temperature conditions (n = 5–6/group). Solid bar represents dark cycle; Open bar represents light cycle. Mean ± SEM. *p < 0.05 vs. *ob/ob* veh; #p < 0.05 vs. 22 °C.

Wild-type mice, but not *ob/ob* mice, are reported to display a negative relationship between energy intake and ambient temperature [8]. Thus our finding that energy intake in vehicle-treated *ob/ob* mice housed at 30 °C was reduced relative to 22 °C during both dark and light cycles was unexpected. While the reason for the discrepancy is unclear, we also found that vehicle-treated *ob/ob* mice failed to increase their energy intake when exposed to 14 °C (Figure 4), despite increasing their energy expenditure, thereby resulting in greater weight loss. These results are consistent with recently published data [8]. Interestingly, systemic leptin replacement reduced energy intake of *ob/ob* mice (relative to vehicle-treated controls) housed at each ambient temperature, including at thermoneutrality (Figure 4). Thus, physiological leptin replacement reduces energy intake even under cool temperature conditions, despite increases of both energy expenditure and core body temperature, and therefore results in greater weight loss than occurs in vehicle-treated *ob/ob* mice.

### 3.3 Effect of physiological leptin replacement on thermoeffector mechanisms at different ambient temperatures

**Figure 5:**
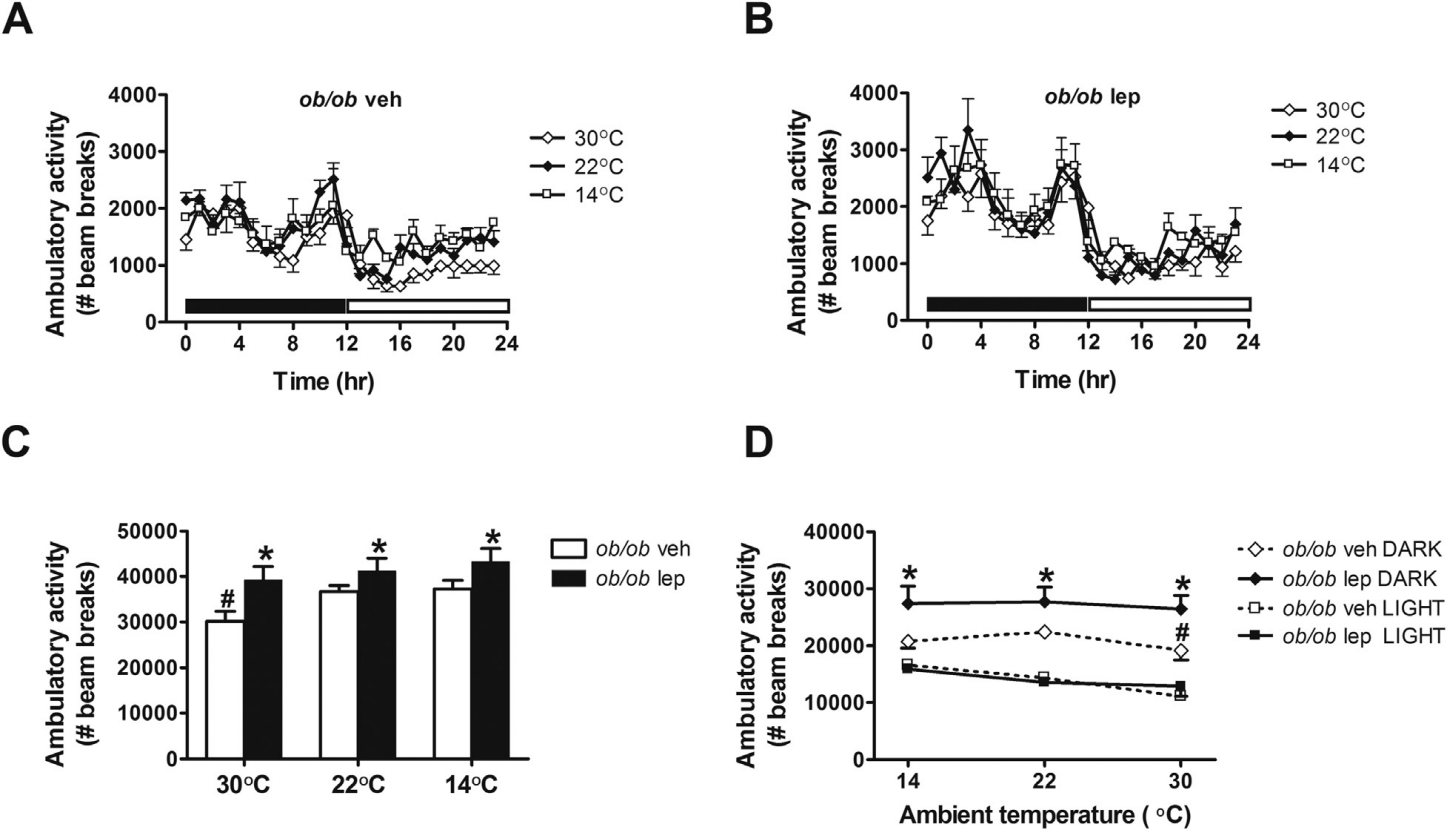
**Effect of leptin treatment on ambulatory at different ambient temperatures**. (**A, B**) Photoperiod-averaged ambulatory activity, (**C**) mean daily ambulatory and (**D**) the relationship between ambulatory activity and ambient temperature during the dark cycle and light cycle in adult male *ob/ob* mice treated with either vehicle or a physiological replacement dose of leptin administered systemically under different ambient temperature conditions (n = 5–6/group). Solid bar represents dark cycle; Open bar represents light cycle. Mean ± SEM. *p < 0.05 vs. *ob/ob* veh; #p < 0.05 vs. 22 °C.

**Figure 6:**
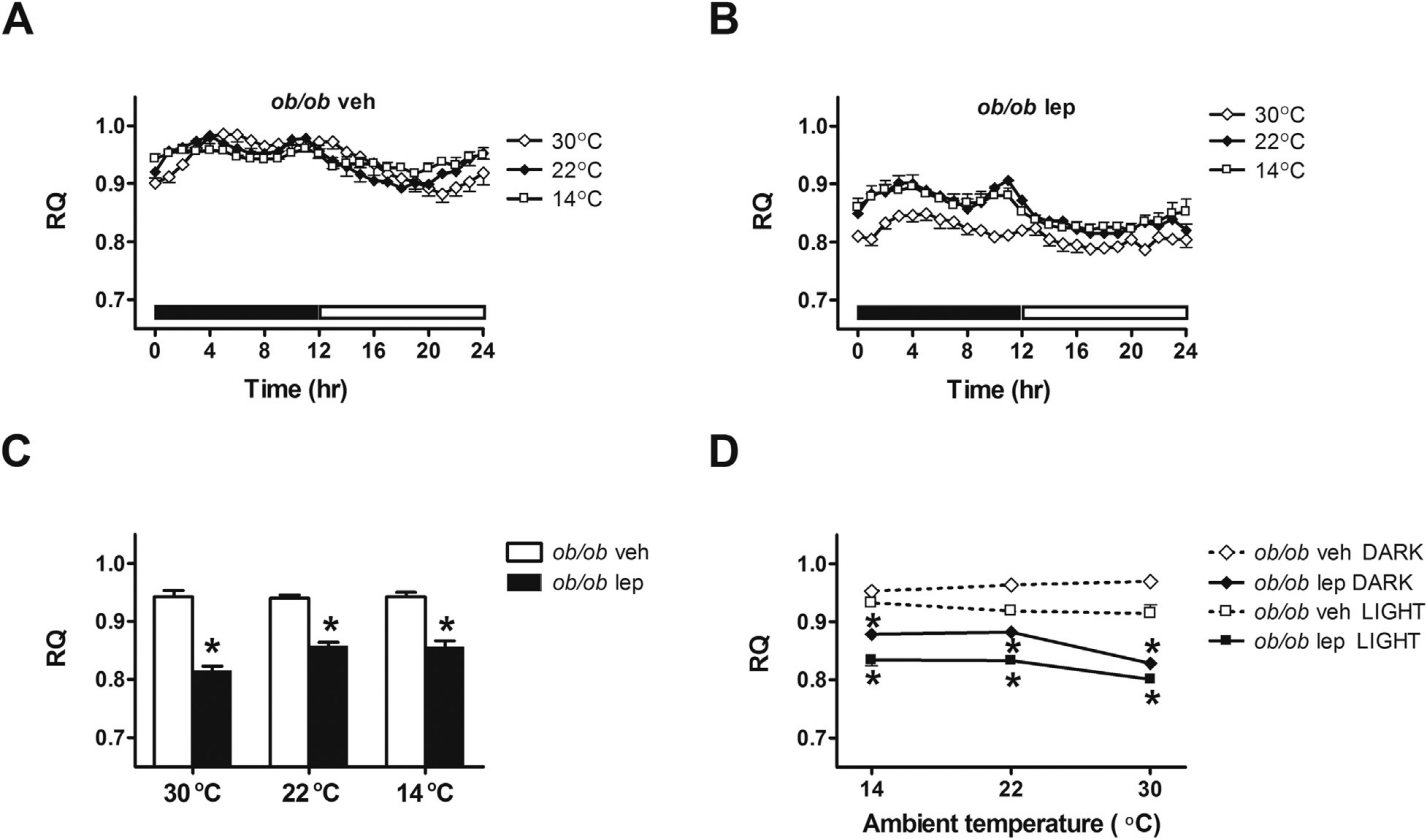
**Effect of leptin treatment on respiratory quotient at different ambient temperatures**. (**A, B**) Photoperiod-averaged respiratory quotient (RQ), (**C**) mean RQ and (**D**) the relationship between RQ and ambient temperature during the dark cycle and light cycle in adult male *ob/ob* mice treated with either vehicle or a physiological replacement dose of leptin administered systemically under different ambient temperature conditions (n = 5–6/group). Solid bar represents dark cycle; Open bar represents light cycle. Mean ± SEM. *p < 0.05 vs. *ob/ob* veh; #p < 0.05 vs. 22 °C.

Despite their ability to increase energy expenditure in response to cold stress, *ob/ob* mice nevertheless exhibit a profound thermoregulatory defect that is partially rescued by physiological leptin replacement. This increase of energy expenditure is unlikely to be explained by changes in ambulatory activity, since activity levels did not change in *ob/ob*-vehicle treated mice when housed at room temperature relative to cool conditions (Figure 5). While ambulatory activity levels were increased in leptin-relative to vehicle-treated *ob/ob* mice at each ambient temperature, again, we observed no change in activity levels among leptin-treated mice housed at 22 °C relative to 14 °C (Figure 5). Thus, changes of ambulatory activity are unlikely to play a major role in the adaptive increase of energy expenditure exhibited by *ob/ob* mice subjected to cold environments. However, consistent with previous reports [25], leptin treatment lowered RQ, indicative of increased fat oxidation in *ob/ob* mice relative to vehicle-treated controls, an affect observed at each different ambient temperature (Figure 6).

**Figure 7:**
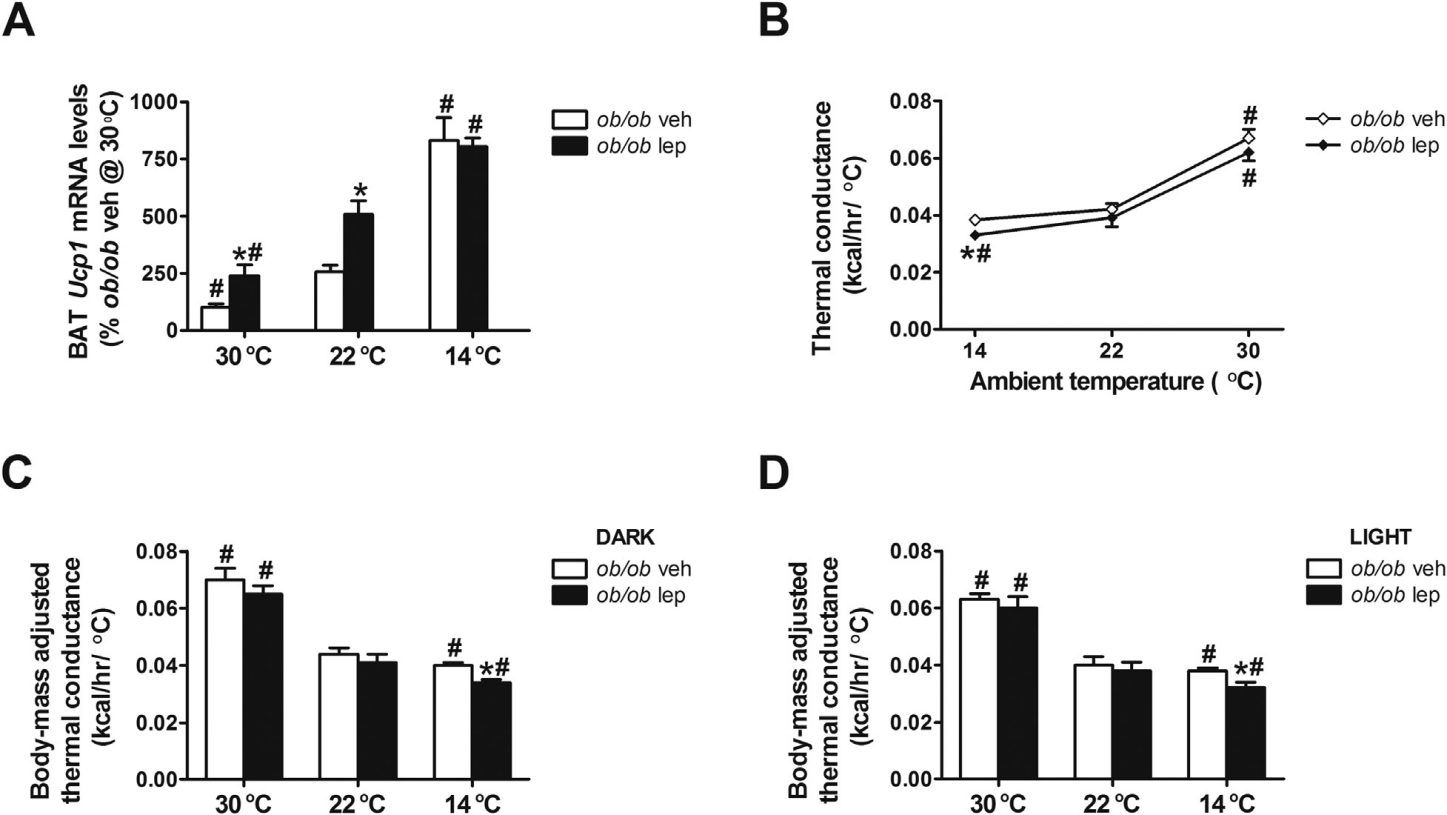
**Effect of ambient temperature and leptin treatment on thermogenic gene expression in brown adipose tissue and on thermal conductance**. Expression levels of uncoupling protein-1 (Ucp1) in (**A**) brown adipose tissue (BAT). (**B**) Whole body thermal conductance (calculated from mean 24 h energy expenditure vs. ambient temperature during the first 24 h of treatment) and (**C, D**) body-mass adjusted whole body thermal conductance during the dark and light cycle in leptin-deficient *ob/ob* mice treated with either vehicle or a physiological replacement dose of leptin (n = 5–6/group). Mean ± SEM. *p < 0.05 vs. *ob/ob* veh; #p < 0.05 vs. 22 °C.

To gain additional insight into the biochemical mechanism(s) underlying increased energy expenditure in cold-exposed *ob/ob* mice, we examined BAT thermogenic capacity by measuring *Ucp1* gene expression in this tissue in *ob/ob* mice that were housed at different ambient temperatures and treated with either vehicle or leptin [26]. We found that BAT *Ucp1* mRNA levels in *ob/ob* mice increased in a dose-dependent manner when housed at temperatures below thermoneutrality (Figure 7A). Combined with their marked increase of energy expenditure (relative to ∼thermoneutral conditions), these data provide further evidence that animals housed at room temperature are under a significant thermal stress [9], [19], [27]. Consistent with previous reports [28], [29], [30], we also found that BAT *Ucp1* mRNA levels were increased with leptin treatment relative to vehicle-treated controls when housed at room temperature (Figure 7A). However, the effect of cold-exposure to increase BAT *Ucp1* mRNA levels was comparable in *ob/ob* mice treated with either vehicle or leptin (Figure 7A). These results suggest that leptin is not required for the effect of a cold challenge (14 °C) to increase BAT thermogenesis [9] and point to an apparent dissociation between the failure of *ob/ob* mice to maintain core body temperature when housed at 14 °C and their apparently intact ability to increase both energy expenditure and BAT thermogenesis.

Effective thermoregulation entails regulatory adjustments of heat loss as well as heat production. The former can be assessed by calculating whole-body thermal conductance (a measure of the ease with which heat flows from the animal to the environment) across different thermal environments. Relative to thermoneutral conditions, thermal conductance decreased progressively with lower ambient temperatures, indicative of reduced heat conservation response loss at lower ambient temperatures (Figure 7B). Moreover, we found that unadjusted thermal conductance was reduced by physiological leptin replacement in *ob/ob* mice exposed to the cold during the first 24 h of treatment (Figure 7B). In addition, it is important to consider that body size is an important determinant of body surface area and hence of whole-body thermal conductance [31] and leptin treatment significantly reduced body weight in *ob/ob* mice over time (Figure 2). Using graphical analysis and linear mixed model analysis, we further revealed that dark cycle, light cycle and average 24 h conductance estimates were well predicted by a mixed factorial model that included body mass (p < 0.0001) and the body mass by ambient temperature interaction term (p < 0.0001). A similar result was obtained when the analysis incorporated an estimate of body surface area, body mass raised to the 2/3 power [31]. Adding group membership to the mixed model analyses revealed a trend for the leptin-treated *ob/ob* mice to have lower body size-adjusted conductance than vehicle controls (e.g., using body mass as the covariate, dark cycle conductance was 0.051 ± 0.002 (vehicle) vs. 0.047 ± 0.002 (leptin) kcal/h/°C; p = 0.06; see Supplemental Table 1). Investigation of this effect by analysis of covariance conducted within each ambient temperature condition revealed a similar reduction of both body mass-adjusted and surface area-adjusted conductance estimates for leptin, relative to vehicle-treated mice at 14 °C (p ≤ 0.005), but not at 22 °C or 30 °C (p ≤ 0.15) (Figure 7C,D). Analogous adjusted analyses of energy expenditure revealed no tendency for the groups to differ in energy expenditure (p ≥ 0.39). Accordingly, physiological leptin replacement in *ob/ob* mice favors a restoration of normothermia during cold exposure by reducing thermal conductance rather than by increasing energy expenditure alone.

## 4 Discussion

The observations that leptin-deficient mice are prone to hypothermia and that this defect is remedied by leptin administration point to a key role for leptin in thermoregulation. To date, however, little is known regarding the physiological role played by leptin in the control of heat production and dissipation. To address this issue, we investigated the effect of a physiological replacement dose of leptin on core body temperature and comprehensive measures of heat production, thermal conductance and energy balance in leptin-deficient *ob/ob* mice exposed to different ambient temperatures. Complimenting and extending previous observations [7], [8], [9], [32], we observed intact and equivalent increases of energy expenditure in both leptin and vehicle-treated *ob/ob* mice exposed to cool environments, along with a similar increase of *Ucp1* gene expression in BAT. These findings indicate that leptin is not required for cold-induced increases of energy expenditure or activation of BAT. Yet the effect of cold exposure to induce hypothermia in leptin-deficient *ob/ob* mice was substantially corrected by physiological leptin replacement. While these observations confirm previous evidence of a role for leptin signaling in thermoregulation [2], they also emphasize that this effect cannot be simply explained by changes of thermogenesis. Instead, our data strongly suggest that leptin plays a physiological role to reduce thermal conductance, and that hypothermia in the setting of leptin deficiency is likely attributable, at least in part, to excessive dissipation of heat to the environment. We also report that systemic leptin replacement failed to correct the disrupted relationship between energy intake and ambient temperature in *ob/ob* mice. Our finding that reduced thermal conductance contributes to the effect of leptin replacement to restore normothermia to cold-exposed *ob/ob* mice offers direct evidence of leptin's physiological role played in the control of this key aspect of thermoregulation.

Previous work demonstrates that wild-type mice are remarkably adept at maintaining both normothermic core body temperature and stable body fat mass when housed at different ambient temperatures [8]. This outcome is achieved by rapidly and precisely adjusting energy intake to match the changing requirements for energy expenditure to meet thermogenic needs [7], [8], [32]. The resultant inverse relationship between energy intake and ambient temperature characteristic of normal animals is disrupted in leptin-deficient *ob/ob* mice, which fail to both increase energy intake and to maintain their core body temperature when confronted with cold exposure. As a result, both lean and fat mass are rapidly depleted to meet the energy costs in a failed effort to maintain euthermia [8]. Our current findings show that physiological leptin replacement reduces energy intake of *ob/ob* mice irrespective of ambient temperature and thus does not protect them from cold-induced weight loss, even though it confers substantial protection against the associated hypothermia. These findings raise the possibility that the relationship between energy intake and ambient temperature may be impaired in other models of obesity, including in high fat (HF)-fed, diet-induced obese (DIO) rodent models, since they are characterized by leptin resistance [33]. In support of this possibility, HF-fed mice gain more body weight and greater body adiposity when housed under thermoneutral conditions compared to room temperature conditions [34], [35], consistent with the notion that obesity pathogenesis involves a defect in the ability to switch off the component of thermoregulatory feeding that was obviated by the imposition of thermoneutrality. Conversely, recent evidence suggests that unlike chow-fed mice, DIO mice housed at temperatures below ∼thermoneutrality preferentially rely on mobilizing their energy stores rather than increasing food intake, and they consequently lose body weight [36]. Common forms of obesity may therefore involve dysregulated coupling of energy homeostasis to thermoregulation.

While the normal compensatory relationship between energy intake and ambient temperature is disrupted in *ob/ob* mice when exposed to the cold, the life-preserving compensatory relationship that exists between energy expenditure and ambient temperature in wild-type mice is preserved in *ob/ob* mice [7], [8], [9]. Specifically, when wild-type or *ob/ob* mice are housed at conditions below thermoneutrality (i.e., room temperature (22 °C)), energy expenditure is markedly increased, indicating that these mice are already under a significant thermal stress. Moreover, recent work suggests that relative to thermoneutral conditions where basal metabolic rate (BMR) comprises ∼60% of total energy expenditure, BMR represents ∼30% of total energy expenditure at room temperature and ∼20% of total energy expenditure at 4 °C [19]. Accordingly, cold-induced thermogenesis comprises of ∼45% of total energy expenditure at 22 °C, and increases further to ∼60% at 4 °C [19]. These findings emphasize the potential impact of housing temperature when analyzing and interpreting obesity phenotypes in mice and attempting to translate these observations to human obesity [27], [37], [38], [39].

Despite increasing energy expenditure, we found that consistent with previous observations [8], [23], *ob/ob* mice are unable to maintain normothermic core body temperature when housed at room temperature and become even more hypothermic when exposed directly to colder conditions. These findings contrast with a recent report suggesting that *ob/ob* mice do not exhibit a further reduction of core body temperature when the magnitude of the cold-stress is increased [9]. A likely explanation for this discrepancy is that the latter studies were conducted using an experimental paradigm in which ambient temperature is gradually but progressively reduced over time (every 2 h using a modified Scholander experiment [9], [40]), whereas our studies involved an immediate and sustained change in ambient temperature. Combined with evidence that *ob/ob* mice become more tolerant of cold stress after they've been cold-adapted [4], these findings collectively suggest that over time, leptin-independent mechanisms can be engaged to compensate for the deleterious effect of leptin deficiency on thermoregulation.

The finding that the hypothermic phenotype of *ob/ob* mice at sub-thermoneutral temperatures is partially corrected by a physiological replacement dose of leptin implies that leptin must either increase energy expenditure, reduce thermal conductance, or both. Our data show that in *ob/ob* mice housed at room temperature, leptin induces a small but significant increase of energy expenditure that was accompanied by increased *Ucp1* mRNA levels in BAT [28], [29], [30]. This finding is at odds with recent evidence that leptin fails to increase energy expenditure in *ob/ob* mice housed at the same temperature [9]. This discrepancy could potentially arise from differences in the route of leptin administration (i.e., twice daily i.p. injections vs. a continuous sc infusion protocol that replaces plasma leptin levels to a physiological level), in indirect calorimetry equipment [41], or both. Nevertheless, both studies confirmed prior evidence that leptin treatment prevents the effect of weight loss to reduce energy expenditure [42], [43]. While additional studies are needed to determine if difference of food intake between leptin- and vehicle-treated animals impacted energy expenditure, the issue is complicated by the observation that reduced food availability predisposes to torpor in *ob/ob* mice [44], and this effect is prevented by leptin [45], despite its inhibitory effect on food intake.

In contrast, however, we observed no effect of physiological leptin replacement on either energy expenditure or *Ucp1* mRNA levels in BAT when *ob/ob* mice were housed at 14 °C. Thus, cold-induced increases of energy expenditure and activation of BAT do not appear to require leptin signaling. This finding seems surprising given evidence that 1) cold-induced activation of Ucp1 plays an important role in adaptive thermogenesis [46], [47], and 2) that *ob/ob* mice deficient in UCP1 fail to increase energy expenditure and survive at temperatures below 12 °C, unlike *ob/ob* littermate controls [48]. However, the findings that cold-adapted Ucp1 deficient mice are able to survive at ambient temperatures as low as 4 °C [46], [47] and that leptin treatment increases energy expenditure in leptin-deficient mice that lack UCP1 (*ob/ob.Ucp1−/−* mice) that enables them to survive the cold [48], suggest that at least some of leptin's action to increase both energy expenditure and body temperature involves mechanisms independent of BAT activation.

Since physiological leptin replacement raised core body temperature of *ob/ob* mice house at 14 °C without increasing energy expenditure, it follows that increased heat conservation must contribute to this effect. Indeed, we report that whole body thermal conductance, a measure of how easily heat flows from the body to the environment, is increased in *ob/ob* relative to wild-type mice across an array of ambient temperatures [8], and that this defect is remedied by administration of leptin at a dose that restores physiological plasma levels. Specifically, we report that physiological leptin replacement reduced body mass- and body surface-adjusted thermal conductance relative to vehicle-treated controls, indicative of reduced heat loss. We interpret these findings to suggest that the effect of leptin to restore normothermia during cold exposure is due, at least in part, to its effect to reduce thermal conductance. While we did not measure heat flux across the body surface, this effect may involve an action of leptin to reduce tail temperature [9], as dissipation of heat from tail (by vasomotor control of tail blood vessels) plays an important role in rodent thermoregulation [1].

This interpretation of leptin's role in thermoregulation differs from the recently reported notion that leptin acts instead by raising the defended level of core body temperature by increasing the core temperature threshold for activating thermogenesis [9]. This is an interesting possibility, but we note that even if it proves to be correct, Newton's law of cooling as applied to thermoregulation (in which the heat loss rate is proportional to the difference in core and ambient temperature [1], [49]) stipulates that an increase of core temperature at any given ambient temperature must involve an increase of either energy expenditure (i.e., heat production) or heat conservation. Since available data indicate that leptin is not required for the increase of energy expenditure exhibited by *ob/ob* mice exposed to the cold, and yet leptin treatment increases core body temperature [9], it follows that leptin must act, at least in part by activating heat conservation mechanisms, i.e., that leptin acts to lower conductance, consistent with our current findings. Future studies are warranted to better understand the mechanisms underlying leptin's action to reduce thermal conductance and thereby to raise core temperature in *ob/ob* mice. We speculate that leptin action in the central nervous system plays a role [2], [50], perhaps via a mechanism related to that recently shown for thyroid hormone in the control of heat conservation [51]. Indeed, *ob/ob* mice are thyroid hormone-deficient, and physiological leptin replacement raises circulating thyroid hormone levels, which may contribute to its effects to enhance heat conservation.

Although leptin did increase ambulatory activity in *ob/ob* mice relative to vehicle-treated controls at different ambient temperatures, the effect was small, and more importantly, activity levels did not change in leptin-treated animals under different temperature environments. Thus leptin-mediated changes in physical activity do not appear to play a major role in the defense of body temperature. In contrast, RQ was reduced by leptin replacement at each temperature, indicative of increased fatty acid oxidation, although this effect could have been confounded by the associated leptin-mediated reduction of food intake.

## 5 Conclusions

In conclusion, we report that physiological leptin levels play a role in conferring the ability to defend normothermia during cold exposure. This effect does not stem from increased energy expenditure, the induction of BAT, or increased ambulatory activity levels, but is due, in part, to an effect of leptin to reduce heat loss. Collectively, these findings identify a new physiological role for leptin as a mediator of reduced heat dissipation elicited by cold exposure, an efficient and economical homeostatic strategy for maintenance of core temperature in light of the extremely high metabolic cost of matching energy intake to markedly elevated energy expenditure levels during cold exposure in small mammals.
