# Supplementary material for: Physiological role for leptin in the control of thermal conductance
Source: Mol Metab. 2016 Jul 20;5(10):892–902. doi: 10.1016/j.molmet.2016.07.005 (PMC5034509; doi:10.1016/j.molmet.2016.07.005)
Supplement: Supplementary file 1 [file mmc1.pdf]

Supplemental Table 1. Body-mass adjusted thermal conductance in leptin and vehicle-treated *ob/ob* mice at different ambient temperatures.

Ambient Temp (°C)	Photocycle	Treatment	Conductance (kcal/h/°C)	SEM	P within Ambient temp.[a]	P mixed model[b]
30	24 h	VEH	0.069	0.004		
30	24 h	LEP	0.061	0.005	0.256	0.075
22	24 h	VEH	0.042	0.002		
22	24 h	LEP	0.038	0.002	0.148	
14	24 h	VEH	0.038	0.001		
14	24 h	LEP	0.038	0.001	0.003	
30	DARK	VEH	0.071	0.004		
30	DARK	LEP	0.064	0.004	0.216	0.060
22	DARK	VEH	0.044	0.002		
22	DARK	LEP	0.040	0.002	0.164	
14	DARK	VEH	0.039	0.001		
14	DARK	LEP	0.036	0.001	0.028	
30	LIGHT	VEH	0.065	0.005		
30	LIGHT	LEP	0.057	0.006	0.338	0.110
22	LIGHT	VEH	0.040	0.001		
22	LIGHT	LEP	0.037	0.002	0.144	
14	LIGHT	VEH	0.037	0.001		
14	LIGHT	LEP	0.034	0.001	0.003	

[a] P for comparison between leptin and vehicle groups within ambient temperature adjusted for body mass.

[b] P from linear mixed model analysis adjusted for body mass, ambient temperature, and the body mass by ambient temperature interaction
