# Supplementary material for: Physiological role for leptin in the control of thermal conductance
Source: Mol Metab. 2016 Jul 20;5(10):892–902. doi: 10.1016/j.molmet.2016.07.005 (PMC5034509; doi:10.1016/j.molmet.2016.07.005)
Supplement: Supplementary file 2 [file mmc2.pdf]

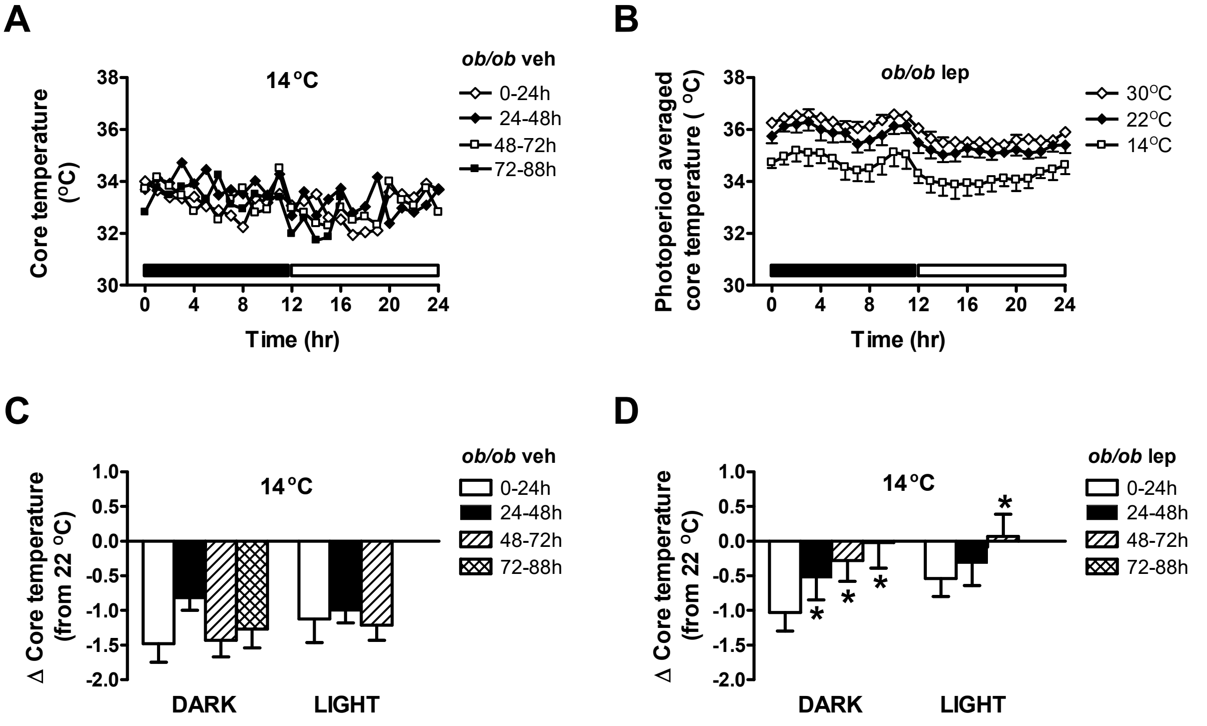

Supplemental Figure 1. Time-course effect of leptin treatment on core body temperature during cold exposure.

(**A, C**) Core body temperature and (**B, D**) the change in core body temperature from 22°C in cold exposed leptin-deficient *ob/ob* mice treated with either vehicle or leptin over time (n=5-6/group). Mean ± SEM. * $p<0.05$ vs. 0-24h.
